# Bifunctionalized Allenes. Part XIII. A Convenient and Efficient Method for Regioselective Synthesis of Phosphorylated α-Hydroxyallenes with Protected and Unprotected Hydroxy Group

**DOI:** 10.3390/molecules19056309

**Published:** 2014-05-16

**Authors:** Ismail E. Ismailov, Ivaylo K. Ivanov, Valerij Ch. Christov

**Affiliations:** Department of Organic Chemistry & Technology, Faculty of Natural Sciences, Konstantin Preslavsky University of Shumen, 115, Universitetska Str., BG-9712 Shumen, Bulgaria; E-Mails: ismail78@mail.bg (I.E.I.); iivanov@shu-bg.net (I.K.I.)

**Keywords:** synthesis, hydroxy group protection, [2,3]-sigmatropic rearrangement, phosphorylated α-hydroxyallenes

## Abstract

The paper describes a convenient and efficient method for regioselective synthesis of phosphorylated α-hydroxyallenes using an atom economical [2,3]-sigmatropic rearrangement of intermediate propargyl phosphites or phosphinites. These can be readily prepared via reaction of protected alkynols with dimethyl chlorophosphite or chlorodiphenyl phosphine respectively in the presence of a base.

## 1. Introduction

The synthesis and application of allene derivatives has had a great influence in preparative organic chemistry during the last three decades. The crucial structural characteristic of allenes is the presence of two π electron clouds separated by a single sp-hybridized carbon atom. Due to that very unique structural and electronic arrangement allenic compounds have an extraordinary reactivity profiles [1,2,3,4,5,6,7,8]. Moreover, functionalized allenes have also attracted growing attention due to their versatility as key building blocks for organic synthesis. The synthetic potential of functionalized allenes has been thoroughly explored in recent years. The research in that area has led to the development of novel methods for the construction of a variety of functionalized heterocyclic and carbocyclic systems [9,10,11,12,13].

There are variety of methods for the construction of hydroxyallenes that include prototropic rearrangement of propargylic alcohols [14,15,16], metal-catalyzed nucleophilic addition of propargylic derivatives to aldehydes [17,18,19,20,21,22,23,24], Cu(I)-catalyzed reaction of propargylic chlorides with Grignard reagents [25,26,27], metal-catalyzed reaction of propargylic oxiranes with organometallic compounds [28,29,30,31,32,33,34,35] and ketones [36,37], reduction of alcohols, ethers, oxiranes *etc.* with aluminium reagents [38,39,40], Pd(0)-catalyzed reaction of cyclic carbonates with acetylenic compounds [41,42], S_N_2’ [43,44] and A_N_ [45,46,47] reactions of metalled alkoxy-allenes with oxiranes and ketones [5], and other routes [48,49].

In addition there are methods [50,51,52,53] for the synthesis of phosphorus-containing allenes (phosphonates [54,55,56,57,58,59], phosphinates [60,61], and phosphine oxides [62,63,64,65,66,67,68,69]) including reactions of α-alkynols with chloride-containing derivatives of phosphorus acids followed by [2,3]-sigmatropic rearrangement. Several diethylphosphono-substituted α-allenic alcohols were prepared by Brel [70,71] directly from alcohols by Horner-Mark rearrangement of unstable propargylic phosphites.

Since the reversible interconversion of propargylic phosphites, phosphonites and phosphinites to allenyl phosphonates, phosphinates and phosphine oxides was discovered five decades ago [60,61], it has become one of the most thoroughly investigated and synthetically applied [2,3]-sigmatropic rearrangements. Numerous synthetic applications of the rearrangement have been reported, such as its use in the synthesis of allenic steroids for substrate-induced inactivation of aromatase [72], in the efficient synthesis of (2*R*)-2-amino-5-phosphonopentanoic acid (AP5) as a powerful and selective *N*-methyl-d-aspartate (NM*D*A) antagonist [73], in the preparation of the phosphonate analogues of phosphatidyl derivatives [74,75], and, in the synthesis of new acyclic analogues of nucleotides containing a purine or pyrimidine moiety and an allenic skeleton [76,77].

Our research program on the chemistry of the bifunctionalized allenes requires a convenient method to introduce a phosphorus-containing group such as phosphonate or phosphine oxide group as well as a hydroxyalkyl group in the first position to the allenic system of double bonds. The above-mentioned groups attract more and more researchers’ attention as useful functionalities in organic synthesis. The emphasis is particularly on the applications of these groups as temporary transformers of chemical reactivity of the allenic system in the synthesis of eventually heterocyclic compounds.

Our scientific interest on the synthesis [78] and electrophilic cyclization reactions [79] of bifunctionalized allenes reported in our previous articles let to the discovery of a convenient and efficient method for regioselective synthesis of phosphorylated α-hydroxyallenes by an atom economical [2,3]-sigmatropic rearrangement of the mediated 4-(tetrahydro-2*H*-pyran-2-yloxy)-propargyl phosphites or phosphinites.

## 2. Results and Discussion

We based our strategy for the synthesis of the phosphorylated α-hydroxyallenes on our experience in preparation of the 4-heteroatom-functionalized allenecarboxylates [78] and relied on the well-precedented [2,3]-sigmatropic shift of propargylic phosphites to allenephosphonates [54,55,56,57,58,59] and propargylic phosphinites to allenyl phosphine oxides [62,63,64,65,66,67,68,69]. We were aware of the fact that a precedent exists for such an approach to the synthesis of the diethylphosphono-substituted α-allenic alcohols [70,71], but as far as we know, a general useful method for regioselective synthesis of phosphorylated (phosphonates and phosphine oxides) α-hydroxyallenes (primary, secondary or tertiary alcohols) with protected or unprotected hygroxy group has not been reported yet.

### 2.1. Synthesis of Phosphorylated α-Hydroxyallenes with Protected Hydroxy Group

The main target in our research, and namely 1,1-bifunctionalized allenes, was achieved as a range of the phosphorylated α-hydroxyallenes **7**, **9**, **10**, and **11** were prepared by applying the following four-step procedure: (i) protection of hydroxy group in the propagylic alcohols **1**; (ii) subsequent reaction with Grignard reagent to give the protected alkynols **5**; (iii) interaction with dimethyl chlorophosphite or chlorodiphenyl phosphine in the presence of a base; and finally (iv) [2,3]-sigmatropic rearrangement of the protected propargyl phosphites or phosphinites.

#### 2.1.1. Synthesis of (Tetrahydro-2*H*-pyran-2-yloxy)-alkynols

**Scheme 1:**
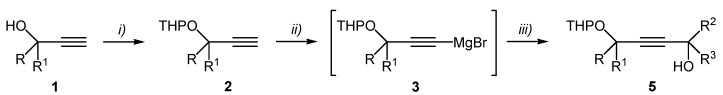
Synthesis of the (tetrahydro-2*H*-pyran-2-yloxy)-alkynols **5**.

The first step in our investigation was to examine the hydroxy group protection in the propargylic alcohols **1** with 3,4-dihydro-2*H*-pyran (DHP) in the presence of pyridinium *p*-toluenesulfonate (PPTS) [80,81,82,83] (Scheme 1 and Table 1). Thus, the formed alkynyloxy-tetrahydro-2*H*-pyrans 2 were isolated by distillation in essentially quantitative yields (95%–99%). Reaction of the protected propargylic compounds **2** with ethyl-magnesium bromide and subsequent dropwise addition of propargyl magnesium bromide **3** generated *in situ* to ketones **4** and reflux for 24 h gave the (tetrahydro-2*H*-pyran-2-yloxy)-alkynols **5** which were stable and were isolated by column chromatography in 53%–61% yields.

*Reagents and Conditions*: *(i)* DHP (3,4-dihydro-2*H*-pyran) (1.5 eq), PPTS (0.1 eq), CH_2_Cl_2_, rt, 4 h, distillation; *(ii)* EtMgBr (1 eq), THF, reflux, 2 h; *(iii)* dropwise addition of **3** to R^2^R^3^C=O **4** (2 eq) (R^2^ = Me, R^3^ = Et; R^2^ = Me, R^3^ = Bu; R^2^ + R^3^ = -(CH_2_)_5_-), reflux, 24 h, column chromatography.

**Table 1:** Synthesis of the (tetrahydro-2*H*-pyran-2-yloxy)-alkynols **5**.

Entry	Alcohol	R	R^1^	R^2^	R^3^	Yield ^a^, %
1	**5a**	H	H	Me	Et	61
2	**5b**	H	H	Me	Bu	59
3	**5c**	H	H	-(CH_2_)_5_-		58
4	**5d**	H	Me	Me	Et	57
5	**5e**	H	Me	Me	Bu	56
6	**5f**	H	Me	-(CH_2_)_5_-		56
7	**5g**	Me	Me	Me	Et	54
8	**5h**	Me	Me	Me	Bu	53

^a^ Isolated yields by chromatographic purification.

#### 2.1.2. Synthesis of Dimethyl 1-(Tetrahydro-2*H*-pyran-2-yloxy)-1,2-dienephosphonates

**Scheme 2:**
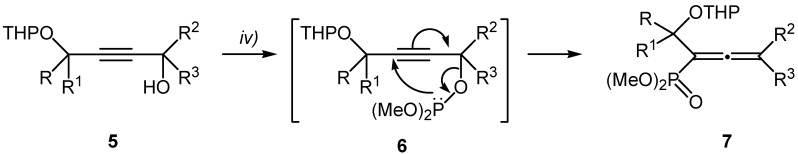
Synthesis of the dimethyl 1-(tetrahydro-2*H*-pyran-2-yloxy)-1,2-dienephosphonates **7**.

Once we had the required propargyl alcohols **5** with protected hydroxyl groups, we were able to investigate the proposed reactions with the corresponding chloro-containing phosphorus reagents such as dimethyl chlorophosphite and chlorodiphenyl phosphine in the presence of a base and subsequent [2,3]-sigmatropic rearrangement of the intermediate 4-(tetrahydro-2*H*-pyran-2-yloxy)-propargyl phosphites or phosphinites **6** and **8**. Let us start with the dimethyl 1-(tetrahydro-2*H*-pyran-2-yloxy)-1,2-dienephosphonates **7a–h** that can be easily prepared via an atom economical 2,3-sigmatropic rearrangement of the 4-(tetrahydro-2*H*-pyran-2-yloxy)-propargyl phosphites **6a–h**, intermediates formed by reaction of the (tetrahydro-2*H*-pyran-2-yloxy)-alkynols **5a–h** with dimethyl chloro-phosphite, prepared *in situ* from phosphorus trichloride and 2 equiv*.* of methanol in the presence of triethylamine, and 2 equiv*.* of pyridine, according to Scheme 2 and Table 2.

*Reagents and Conditions*: (*iv)* PCl_3_(1 eq), Et_3_N (1.1 eq), Et_2_O, −70 °C, 30 min stirring, pyridine (2.2 eq), MeOH (2 eq), Et_2_O, [2,3-σ]-rearrangement, −70 °C, 1 h, rt, 10 h, column chromatography.

**Table 2:** Synthesis of the dimethyl 1-(tetrahydro-2*H*-pyran-2-yloxy)-1,2-dienephosphonates **7**.

Entry	Allene	R	R^1^	R^2^	R^3^	Yield ^a^, %
1	**7a**	H	H	Me	Et	78
2	**7b**	H	H	Me	Bu	75
3	**7c**	H	H	-(CH_2_)_5_-		73
4	**7d**	H	Me	Me	Et	74
5	**7e**	H	Me	Me	Bu	72
6	**7f**	H	Me	-(CH_2_)_5_-		75
7	**7g**	Me	Me	Me	Et	71
8	**7h**	Me	Me	Me	Bu	70

^a^ Isolated yields by chromatographic purification.

#### 2.1.3. Synthesis of 2-[2-(Diphenylphosphinoyl-2,3-dienyloxy)]-tetrahydro-2*H*-pyrans

**Scheme 3:**
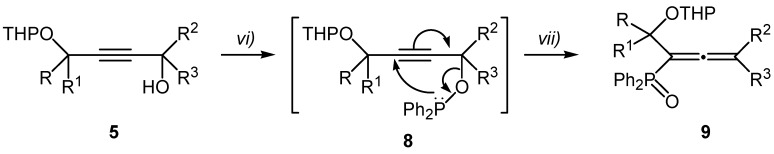
Synthesis of the 2-(2-diphenylphosphinoyl-2,3-dienyloxy)-tetrahydro-2*H*-pyrans **9**.

Next, the reaction of the (tetrahydro-2*H*-pyran-2-yloxy)-alkynols **5a–h** with chlorodiphenyl phosphine in the presence of triethylamine at −70 °C gave the expected 2-(2-diphenylphosphinoyl-2,3-dienyloxy)-tetrahydro-2*H*-pyrans **9a–h** in very good yields (Table 3) as a result of [2,3]-sigmatropic rearrangement of the 4-(tetrahydro-2*H*-pyran-2-yloxy)-propargyl phosphinites **8a–h** for 8 h at room temperature, according to the reaction sequence outlined in Scheme 3.

**Table 3:** Synthesisof the 2-(2-diphenylphosphinoyl-2,3-dienyloxy)-tetrahydro-2*H*-pyrans **9**.

Entry	Allene	R	R^1^	R^2^	R^3^	Yield ^a^, %
1	**9a**	H	H	Me	Et	86
2	**9b**	H	H	Me	Bu	84
3	**9c**	H	H	-(CH_2_)_5_-		81
4	**9d**	H	Me	Me	Et	83
5	**9e**	H	Me	Me	Bu	82
6	**9f**	H	Me	-(CH_2_)_5_-		80
7	**9g**	Me	Me	Me	Et	80
8	**9h**	Me	Me	Me	Bu	78

^a^ Isolated yields by chromatographic purification.

*Reagents and Conditions*: *(vi)* Ph_2_PCl (1 eq), Et_3_N (1.1 eq), Et_2_O, −70 °C; *(vii)* [2,3-σ]-rearrangement, −70 °C, 1 h, rt, 8 h, column chromatography.

A new family of phosphorylated α-hydroxyallenes with protected hydroxyl group **7** and **9** were synthesized via an atom economical and regioselective [2,3]-sigmatropic rearrangement of the intermediate formed propargyl phosphites or phosphinites in the reaction of protected alkynols **5** with dimethylchloro phosphite or chlorodiphenyl phosphine in the presence of triethylamine. 

### 2.2. Synthesis of Phosphorylated α-Hydroxyallenes with Unprotected Hydroxy Group

**Scheme 4:**
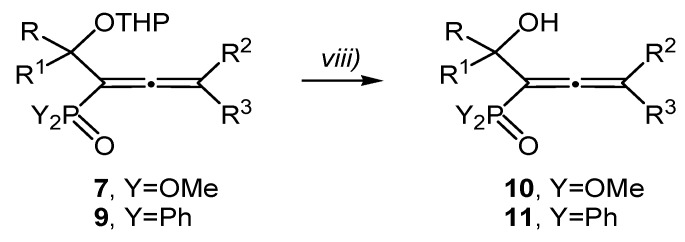
Synthesis of the 1-hydroxyalkyl-1,2-dienephosphonates **10**, the 3-diphenylphosphinoyl-2,3-dien-1-ols **11a**–**c** and the 3-diphenylphosphinoyl-3,4-dien-2-ols **11d**–**h**.

Compounds **7** and **9** were stable enough to be handled at ambient temperature. The hydroxy group was deprotected by stirring the ethanol solution of the protected hydroxylalkyl-allenephosphonates **7** and hydroxylalkyl-allenyl phosphine oxides **9** in the presence of 0.1 equiv. PPTS at room temperature for 6 h, according to Scheme 4 and Table 4.

*Reagents and Conditions*: *(viii)* PPTS (0.1 eq), EtOH, rt, 6 h, stirring.

**Table 4:** Synthesis of the 1-hydroxyalkyl-1,2-dienephosphonates **10**, the 3-diphenylphosphinoyl-2,3-dien-2-ols **11a**–**c** and the 3-diphenylphosphinoyl-3,4-dien-2-ols **11d**–**h**.

Entry	Allene	R	R^1^	R^2^	R^3^	Yield ^a^, %
1	**10a**	H	H	Me	Et	80
2	**10b**	H	H	Me	Bu	78
3	**10c**	H	H	-(CH_2_)_5_-		77
4	**10d**	H	Me	Me	Et	80
5	**10e**	H	Me	Me	Bu	79
6	**10f**	H	Me	-(CH_2_)_5_-		81
7	**10g**	Me	Me	Me	Et	79
8	**10h**	Me	Me	Me	Bu	78
9	**11a**	H	H	Me	Et	86
10	**11b**	H	H	Me	Bu	83
11	**11c**	H	H	-(CH_2_)_5_-		81
12	**11d**	H	Me	Me	Et	87
13	**11e**	H	Me	Me	Bu	85
14	**11f**	H	Me	-(CH_2_)_5_-		88
15	**11g**	Me	Me	Me	Et	84
16	**11h**	Me	Me	Me	Bu	83

^a^ Isolated yields by chromatographic purification.

After a conventional work-up, all allenic products **7**, **9**, **10**, and **11**were isolated as stable yellow or orange oils by column chromatography and identified by ^1^H-, ^13^C-, and ^31^P-NMR and IR spectra as well as by elemental analysis.

## 3. Experimental Section

### 3.1. General Information

All new synthesized compounds were purified by column chromatography and characterized on the basis of NMR, IR, and microanalytical data. NMR spectra were recorded on DRX Bruker Avance-250 (^1^H at 250.1 MHz, ^13^C at 62.9 MHz, ^31^P at 101.2 MHz) and Bruker Avance II + 600 (Bruker BioSpinGmbH, Karlsruhe, Germany) (^1^H at 600.1 MHz, ^13^C at 150.9 MHz, ^31^P at 242.9 MHz) spectrometers for solutions in CDCl_3_. All ^1^H-and ^13^C-NMR experiments were measured referring to the signal of internal TMS and ^3^^1^P-NMR experiments were measured referring to the signal of external 85% H_3_PO_4_. *J* values are given in hertz. IR spectra were recorded with an FT-IRAfinity-1 Shimadzu spectrophotometer (Shimadzu, Tokyo, Japan). Elemental analyses were carried out by the Microanalytical Service Laboratory of Faculty of Chemistry and Pharmacy, University of Sofia, Bulgaria, using Vario EL*3* CHNS(O) (Elementar Analysensysteme, Hanau, Germany). Column chromatography was performed on Kieselgel F_254_ 60 (70–230 mesh ASTM, 0.063–0.200 nm, Merck, Darmstadt, Germany). Et_2_O and THF were distilled from Na wire/benzophenone, CH_2_Cl_2_ was distilled over CaH_2_, other commercially available chemicals were used without additional purification unless otherwise noted. Reactions were carried out in oven dried glassware under an argon atmosphere and exclusion of moisture. All compounds were checked for purity on TLC plates Kieselgel F_254_ 60 (Merck).

### 3.2. General Procedure [80,81,82,83] for Synthesis of the Alkynyloxy-tetrahydro-2H-pyrans **2**

A solution of alkynols **1** (60.0 mmol) and DHP (7.6 g, 90.0 mmol) [0.152 g/mL] in dry methylene chloride (50 mL) containing PPTS (1.5 g, 6.0 mmol) [0.03 g/mL] is stirred for 4 h at room temperature. Then the reaction was quenched with saturated NaHCO_3_ end extracted with methylene chloride. The organic layer was dried over anhydrous sodium sulfate. After evaporation of the solvent, distillation gives an essentially quantitative yield of the alkynyloxy-tetrahydro-2*H*-pyrans **2** (95%–99%) which are described in the literature [80,81,82,83].

### 3.3. General Procedure for Synthesis of (Tetrahydro-2H-pyran-2-yloxy)-alkynols **5**

Ethylmagnesium bromide [prepared from magnesium (1.2 g, 50.0 mmol) [0.024 g/mL] and ethyl bromide (5.5 g, 50.0 mmol) [0.11 g/mL] in dry THF (50 mL)] is added dropwise under stirring to substituted alkynyloxy-tetrahydro-2*H*-pyrans **2** (50.0 mmol) and then the mixture is refluxed for 2 h. The solution of the prepared alkynyl magnesium bromides **3** is added dropwise under stirring to the ketones **4** (100.0 mmol). The mixture is refluxed for 24 h and after cooling is hydrolyzed with a saturated aqueous solution of ammonium chloride. The organic layer is separated, washed with water, and dried over over anhydrous sodium sulfate. Solvent and the excess of ketone are removed by distillation. Purification of the residue is achieved by column chromatography (silica gel, Kieselgel Merck 60 F_254_) with ethyl acetate-hexane (5:1). The pure products **5** had the following properties:

*3-Methyl-6-(tetrahydro-2H-pyran-2-yloxy)-hex-4-yn-3-ol* (**5a**). Colourless oil, yield: 61%. R_f_ 0.53; IR (neat, cm^−1^): 1121 (C-O-C), 3439 (OH). ^1^H-NMR (600.1 MHz): δ 1.37 (t, *J =* 7.2 Hz, 3H, Me-CH_2_), 1.40 (s, 3H, Me-C-OH), 1.48, 1.67, 1.71, 3.59, 4.62 (overlapping multiplets, 9H, OTHP), 1.79 (m, 2H, Me-CH_2_), 3.54 (s, 1H, OH), 4.29 (m, 2H, CH_2_O). ^13^C-NMR (150.9 MHz) δ = 9.9, 19.0, 26.1, 28.4, 30.7, 37.5, 55.0, 62.3, 66.2, 81.5, 89.4, 97.7. Anal. Calcd for C_12_H_20_O_3_ (212.29): C 67.89, H 9.50. Found: C 67.81, H 9.44.

*4-Methyl-1-(tetrahydro-2H-pyran-2-yloxy)-oct-2-yn-4-ol* (**5b**). Colourless oil, yield: 59%. R_f_ 0.49; IR (neat, cm^−1^): 1121 (C-O-C), 3420 (OH). ^1^H-NMR (250.1 MHz): δ 0.87 (t, *J =* 6.5 Hz, 3H, Me-(CH_2_)_3_), 1.39 (s, 3H, Me-C-OH), 1.34–1.39, 1.48–1.80, 3.61, 4.72 (overlapping multiplets, 15H, OTHP + (CH_2_)_3_-Me), 2.70 (s, 1H, OH), 4.24 (m, 2H, CH_2_O). ^13^C-NMR (62.9 MHz) δ = 14.7, 19.2, 23.9, 24.7, 25.6, 29.4, 30.8, 45.5, 55.3, 60.9, 64.2, 80.1, 89.0, 97.1. Anal. Calcd for C_14_H_24_O_3_ (240.34): C 69.96, H 10.07. Found: C 70.03, H 10.12.

*1-[3-(Tetrahydro-2H-pyran-2-yloxy)-prop-1-ynyl]-cyclohexanol* (**5c**). Colourless oil, yield: 58%. R_f_ 0.48; IR (neat, cm^−1^): 1120 (C-O-C), 3412 (OH). ^1^H-NMR (250.1 MHz): δ 1.30–1.77, 1.96–2.01, 2.10–2.16, 3.54–3.72, 4.70–4.74 (overlapping multiplets, 19H, OTHP + (CH_2_)_5_), 3.51 (s, 1H, OH), 4.27 (m, 2H, CH_2_O). ^13^C-NMR (62.9 MHz) δ = 19.2, 23.2, 25.7, 26.1, 30.4, 40.0, 53.8, 62.5, 69.2, 81.0, 88.9, 96.8. Anal. Calcd for C_14_H_22_O_3_ (238.32): C 70.56, H 9.30. Found: C 70.65, H 9.36.

*3-Methyl-6-(tetrahydro-2H-pyran-2-yloxy)-hept-4-yn-3-ol* (**5d**). Colourless oil, yield: 57%. R_f_ 0.54; IR (neat, cm^−1^): 1122 (C-O-C), 3398 (OH). ^1^H-NMR (600.1 MHz): δ 1.36 (t, *J =* 7.4 Hz, 3H, Me-CH_2_), 1.38 (s, 3H, Me-C-OH), 1.46, 1.62, 1.71, 3.62, 4.71 (overlapping multiplets, 9H, OTHP), 1.49 (d, *J =* 7.0 Hz, 3H, Me-CH), 1.67 (m, 2H, Me-CH_2_), 3.24 (s, 1H, OH), 4.78 (m, 1H, CH-Me). ^13^C-NMR (150.9 MHz) δ = 9.4, 20.5, 22.8, 26.2, 28.7, 30.9, 36.4, 61.7, 63.0, 66.9, 84.5, 88.7, 99.1. Anal. Calcd for C_13_H_22_O_3_ (226.31): C 68.99, H 9.80. Found: C 69.06, H 9.75.

*5-Methyl-2-(tetrahydro-2H-pyran-2-yloxy)-non-3-yn-5-ol* (**5e**). Colourless oil, yield: 56%. R_f_ 0.51; IR (neat, cm^−1^): 1123 (C-O-C), 3432 (OH). ^1^H-NMR (600.1 MHz): δ 0.88 (t, *J =* 6.3 Hz, 3H, Me-(CH_2_)_3_), 1.36 (s, 3H, Me-C-OH), 1.33–1.40, 1.46–1.79, 3.76, 4.78 (overlapping multiplets, 15H, OTHP + (CH_2_)_3_-Me), 1.52 (d, *J =* 6.9 Hz, 3H, Me-CH), 2.54 (s, 1H, OH), 4.66 (m, 1H, CH-Me). ^13^C-NMR (150.9 MHz) δ = 14.4, 20.2, 22.4, 24.2, 24.9, 26.1, 30.2, 31.0, 45.4, 62.7, 63.0, 64.4, 84.2, 88.1, 99.4. Anal. Calcd for C_15_H_26_O_3_ (254.37): C 70.83, H 10.30. Found: C 70.87, H 10.23.

*1-[3-(Tetrahydro-2H-pyran-2-yloxy)-but-1-ynyl]-cyclohexanol* (**5f**). Colourless oil, yield: 56%. R_f_ 0.48; IR (neat, cm^−1^): 1119 (C-O-C), 3429 (OH). ^1^H-NMR (250.1 MHz): δ 1.29–1.52, 1.67–1.84, 1.95–2.12, 3.50–3.87, 4.79–4.82 (overlapping multiplets, 19H, OTHP + (CH_2_)_5_), 1.49 (d, *J =* 7.0 Hz, 3H, Me-CH), 3.32 (s, 1H, OH), 4.71 (m, 1H, CH-Me). ^13^C-NMR (62.9 MHz) δ = 20.1, 22.5, 23.0, 24.7, 26.0, 32.4, 40.6, 61.9, 62.4, 68.9, 83.2, 90.2, 98.9. Anal. Calcd for C_15_H_24_O_3_ (252.35): C 71.39, H 9.59. Found: C 71.30, H 9.66.

*3,6-Dimethyl-6-(tetrahydro-2H-pyran-2-yloxy)-hept-4-yn-3-ol* (**5g**). Colourless oil, yield: 54%. R_f_ 0.49; IR (neat, cm^−1^): 1120 (C-O-C), 3416 (OH). ^1^H-NMR (600.1 MHz): δ 1.34 (t, *J =* 7.4 Hz, 3H, Me-CH_2_), 1.38 (s, 3H, Me-C-OH), 1.43, 1.66, 1.70, 3.69, 4.91 (overlapping multiplets, 9H, OTHP), 1.51 (s, 6H, 2Me), 1.68 (m, 2H, Me-CH_2_), 3.22 (s, 1H, OH). ^13^C-NMR (150.9 MHz) δ = 9.3, 21.1, 25.4, 28.6, 30.0, 32.4, 35.7, 64.1, 66.3, 71.0, 82.3, 86.5, 96.4. Anal. Calcd for C_14_H_24_O_3_ (240.34): C 69.96, H 10.07. Found: C 69.89, H 10.15.

*2,5-Dimethyl-2-(tetrahydro-2H-pyran-2-yloxy)-non-3-yn-5-ol* (**5h**). Colourless oil, yield: 53%. R_f_ 0.45; IR (neat, cm^−1^): 1119 (C-O-C), 3421 (OH). ^1^H-NMR (600.1 MHz): δ 0.87 (t, *J =* 6.4 Hz, 3H, Me-(CH_2_)_3_), 1.34 (s, 3H, Me-C-OH), 1.36–1.42, 1.47–1.72, 3.59, 4.89 (overlapping multiplets, 15H, OTHP + (CH_2_)_3_-Me), 1.50 (s, 6H, 2Me), 2.542 (s, 1H, OH). ^13^C-NMR (150.9 MHz) δ = 14.7, 21.1, 24.3, 24.7, 25.7, 28.4, 30.0, 31.7, 44.2, 64.7, 64.9, 71.3, 82.4, 86.1, 96.7. Anal. Calcd for C_16_H_28_O_3_ (268.39): C 71.60, H 10.52. Found: C 71.52, H 10.58.

### 3.4. General Procedure for Synthesis of the Dimethyl 1-(Tetrahydro-2H-pyran-2-yloxy)-1,2-dienephosphonates **7**

To a solution of phosphorus trichloride (2.8 g, 20.0 mmol) [0.047 g/mL] and triethylamine (2.2 g, 22.0 mmol) [0.037 g/mL] in dry diethyl ether (60 mL) at –70 °C was added dropwise with stirring a solution of the (tetrahydro-2*H*-pyran-2-yloxy)-alkynols 5 (20.0 mmol) in the same solvent (20 mL). After 30 min stirring at the same temperature a solution of pyridine (3.1 g, 44.0 mmol) [0.062 g/mL] and of methanol (1.3 g, 40.0 mmol) [0.026 g/mL] in dry diethyl ether (50 mL) were added. The reaction mixture was stirred for an hour at the same temperature and for 10 h at room temperature. The mixture was then washed with water, 2 N HCl, extracted with ether, washed with saturated NaCl, and dried over anhydrous sodium sulfate. After evaporation of the solvent, the residue was chromatographed on a column (silica gel, Kieselgel Merck 60 F_254_) with a mixture of ethyl acetate and hexane (10:1) as eluent to give the pure products 7 as oils, which had the following properties:

*Dimethyl 3-methyl-1-(tetrahydro-2H-pyran-2-yloxymethyl)-penta-1,2-dienephosphonate* (**7a**). Yellow oil, yield: 78%. R_f_ 0.58; IR (neat, cm^−1^): 1119 (C-O-C), 1250 (P=O), 1958 (C=C=C). ^1^H-NMR (600.1 MHz): δ 1.07 (t, *J =* 7.4 Hz, 3H, Me-CH_2_), 1.53, 1.60, 1.71, 3.53, 4.32 (overlapping multiplets, 9H, OTHP), 1.80 (d, *J =* 6.7 Hz, 3H, Me-C=), 2.07 (m, 2H, Me-CH_2_), 3.76 (d, *J =* 11.2 Hz, 3H, MeO), 4.14 (m, 2H, CH_2_O). ^13^C-NMR (150.9 MHz) δ = 12.0 (*J =* 7.6 Hz), 18.1 (*J =* 6.6 Hz), 19.2, 25.5, 26.5 (*J =* 4.2 Hz), 30.4, 52.8 (*J =* 6.2 Hz), 61.9, 64.9 (*J =* 10.1 Hz), 90.7 (*J =* 191.2 Hz), 97.2, 104.6 (*J =* 15.6 Hz), 208.6 (*J =* 5.5 Hz). ^31^P-NMR (242.9 MHz): δ 20.3. Anal. Calcd for C_14_H_25_O_5_P (304.32): C 55.25; H 8.28. Found: C 55.33; H 8.19.

*Dimethyl 3-methyl-1-(tetrahydro-2H-pyran-2-yloxymethyl)-hepta-1,2-dienephosphonate* (**7b**). Yellow oil, yield: 75%. R_f_ 0.59; IR (neat, cm^−1^): 1121 (C-O-C), 1251 (P=O), 1956 (C=C=C). ^1^H-NMR (600.1 MHz): δ 0.90 (t, *J =* 7.2 Hz, 3H, Me-(CH_2_)_3_), 1.44, 1.53, 1.60, 3.53, 4.36 (overlapping multiplets, 9H, OTHP), 1.78 (d, *J =* 6.5 Hz, 3H, Me-C=), 1.36, 1.82, 2.05 (overlapping multiplets, 6H, Me-(CH_2_)_3_), 3.75 (d, *J =* 11.2 Hz, 3H, MeO), 4.09 (m, 2H, CH_2_O). ^13^C-NMR (150.9 MHz) δ = 13.9, 18.0 (*J =* 6.7 Hz), 19.2, 22.2, 25.5, 29.4, 30.3, 32.9, 52.7 (*J =* 6.3 Hz), 61.8, 64.9 (*J =* 10.1 Hz), 90.3 (*J =* 191.7 Hz), 97.3, 102.8 (*J =* 16.2 Hz), 208.8 (*J =* 5.4 Hz). ^31^P-NMR (242.9 MHz): δ 20.4. Anal. Calcd for C_16_H_29_O_5_P (332.37): C 57.82, H 8.79. Found: C 57.90, H 8.72. 

*Dimethyl 2-cyclohexylidene-1-(tetrahydro-2H-pyran-2-yloxymethyl)-ethenephosphonate* (**7c**). Yellow oil, yield: 73%. R_f_ 0.57; IR (neat, cm^−1^): 1118 (C-O-C), 1252 (P=O), 1953 (C=C=C). ^1^H-NMR (600.1 MHz): δ 1.25–2.23, 3.55, 3.86, 4.31 (overlapping multiplets, 19H, (CH_2_)_5_ + OTHP), 3.74 (d, *J =* 11.1 Hz, 3H, MeO), 4.15 (m, 2H, CH_2_O). ^13^C-NMR (150.9 MHz) δ = 19.1, 25.5, 25.7, 26.5, 30.3 (*J =* 5.9 Hz), 30.4, 52.9 (*J =* 6.2 Hz), 62.0, 64.7 (*J =* 10.8 Hz), 88.6 (*J =* 190.7 Hz), 97.2, 105.1 (*J =* 15.6 Hz), 205.6 (*J =* 5.1 Hz). ^31^P-NMR (242.9 MHz): δ 21.2. Anal. Calcd for C_16_H_27_O_5_P (330.36): C 58.17, H 8.24. Found: C 58.24, H 8.18.

*Dimethyl 3-methyl-1-[1-(tetrahydro-2H-pyran-2-yloxy)-ethyl]-penta-1,2-dienephosphonate* (**7d**). Orange oil, yield: 74%. R_f_ 0.44; IR (neat, cm^−1^): 1122 (C-O-C), 1259 (P=O), 1951 (C=C=C). ^1^H-NMR (600.1 MHz): δ 0.95 (t, *J =* 7.3 Hz, 3H, Me-CH_2_), 1.41 (dd, *J =* 6.4 Hz, *J =* 10.2 Hz, 3H, Me-CHO), 1.51, 1.58, 1.68, 3.63, 4.38 (overlapping multiplets, 9H, OTHP), 1.74 (d, *J =* 6.6 Hz, 3H, Me-C=), 2.04 (m, 2H, Me-CH_2_), 3.77 (d, *J =* 11.2 Hz, 3H, MeO), 4.67 (m, 1H, CHO). ^13^C-NMR (150.9 MHz) δ = 12.3 (*J =* 7.5 Hz), 18.5 (*J =* 6.3 Hz), 19.4, 23.4 (*J =* 7.6 Hz), 25.5, 27.7 (*J =* 4.6 Hz), 30.5, 52.5 (*J =* 6.3 Hz), 62.4, 67.4 (*J =* 10.3 Hz), 95.8, 96.4 (*J =* 192.0 Hz), 104.4 (*J =* 15.9 Hz), 209.2 (*J =* 5.1 Hz). ^3^^1^P-NMR (242.9 MHz): δ 20.4. Anal. Calcd for C_15_H_27_O_5_P (318.35): C 56.59, H 8.55. Found: C 56.64, H 8.63.

*Dimethyl 3-methyl-1-[1-(tetrahydro-2H-pyran-2-yloxy)-ethyl]-hepta-1,2-dienephosphonate* (**7e**). Orange oil, yield: 72%. R_f_ 0.43; IR (neat, cm^−1^): 1120 (C-O-C), 1254 (P=O), 1956 (C=C=C). ^1^H-NMR (600.1 MHz): δ 0.93 (t, *J =* 7.1 Hz, 3H, Me-(CH_2_)_3_), 1.43 (dd, *J =* 6.3 Hz, *J =* 10.0 Hz, 3H, Me-CHO), 1.48, 1.55, 1.64, 3.62, 4.38 (overlapping multiplets, 9H, OTHP), 1.77 (d, *J =* 6.6 Hz, 3H, Me-C=), 1.41, 1.74, 2.11 (overlapping multiplets, 6H, Me-(CH_2_)_3_), 3.76 (d, *J =* 11.2 Hz, 3H, MeO), 4.64 (m, 1H, CHO). ^13^C-NMR (150.9 MHz) δ = 13.8, 18.8 (*J =* 6.5 Hz), 19.5, 22.7, 23.5 (*J =* 7.5 Hz), 25.7, 29.6, 30.4, 32.8, 52.3 (*J =* 6.2 Hz), 62.3, 68.6 (*J =* 10.2 Hz), 91.4 (*J =* 191.7 Hz), 95.6, 103.4 (*J =* 16.2 Hz), 209.0 (*J =* 5.3 Hz). ^3^^1^P-NMR (242.9 MHz): δ 20.5. Anal. Calcd for C_17_H_31_O_5_P (346.40): C 58.94, H 9.02. Found: C 59.01, H 8.96.

*Dimethyl 1-cyclohexylidenemethylene-2-(tetrahydro-2H-pyran-2-yloxy)-propanephosphonate* (**7f**). Dark orange oil, yield: 75%. R_f_ 0.42; IR (neat, cm^−1^): 1122 (C-O-C), 1258 (P=O), 1953 (C=C=C). ^1^H-NMR (600.1 MHz): δ 1.31–2.27, 3.57, 3.71, 4.34 (overlapping multiplets, 19H, (CH_2_)_5_ + OTHP), 1.42 (d, *J =* 6.2 Hz, 3H, Me-CHO), 3.74 (d, *J =* 11.1 Hz, 3H, MeO), 4.51 (m, 1H, CHO). ^13^C-NMR (150.9 MHz) δ = 19.6, 23.5 (*J =* 7.6 Hz), 25.6, 24.7, 25.8, 29.4 (*J =* 5.7 Hz), 30.6, 52.8 (*J =* 6.3 Hz), 62.6, 65.8 (*J =* 10.6 Hz), 93.8 (*J =* 189.6 Hz), 94.7, 106.0 (*J =* 15.5 Hz), 204.3 (*J =* 5.0 Hz). ^3^^1^P-NMR (242.9 MHz): δ 20.2. Anal. Calcd for C_17_H_29_O_5_P (344.38): C 59.29, H 8.49. Found: C 59.36, H 8.43.

*Dimethyl 3-methyl-1-[1-methyl-1-(tetrahydro-2H-pyran-2-yloxy)-ethyl]-penta-1,2-dienephosphonate* (**7g**). Orange oil, yield: 71%. R_f_ 0.44; IR (neat, cm^−1^): 1117 (C-O-C), 1252 (P=O), 1949 (C=C=C). ^1^H-NMR (600.1 MHz): δ 1.05 (t, *J =* 7.4 Hz, 3H, Me-CH_2_), 1.45 (d, *J =* 10.3 Hz, 6H, Me_2_CO), 1.47, 1.60, 1.64, 3.68, 4.35 (overlapping multiplets, 9H, OTHP), 1.79 (d, *J =* 6.6 Hz, 3H, Me-C=), 2.06 (m, 2H, Me-CH_2_), 3.76 (d, *J =* 11.3 Hz, 3H, MeO). ^13^C-NMR (150.9 MHz) δ = 12.4 (*J =* 7.6 Hz), 18.4 (*J =* 6.4 Hz), 20.4, 25.3, 31.1 (*J =* 8.1 Hz), 27.7 (*J =* 4.8 Hz), 31.3, 51.9 (*J =* 6.6 Hz), 63.2, 68.4 (*J =* 10.0 Hz), 92.4, 99.4 (*J =* 194.0 Hz), 103.8 (*J =* 15.3 Hz), 208.5 (*J =* 5.0 Hz). ^3^^1^P-NMR (242.9 MHz): δ 21.4. Anal. Calcd for C_16_H_29_O_5_P (332.37): C 57.82, H 8.79. Found: C 57.76, H 8.87.

*Dimethyl 3-methyl-1-[1-methyl-1-(tetrahydro-2H-pyran-2-yloxy)-ethyl]-hepta-1,2-dienephosphonate* (**7h**). Orange oil, yield: 70%. R_f_ 0.42; IR (neat, cm^−1^): 1121 (C-O-C), 1254 (P=O), 1950 (C=C=C). ^1^H-NMR (600.1 MHz): δ 0.91 (t, *J =* 7.2 Hz, 3H, Me-(CH_2_)_5_), 1.49 (d, *J =* 10.4 Hz, 6H, Me_2_CO), 1.42, 1.73, 2.06 (overlapping multiplets, 6H, Me-(CH_2_)_3_), 1.46, 1.57, 1.62, 3.64, 4.37 (overlapping multiplets, 9H, OTHP), 1.78 (d, *J =* 6.6 Hz, 3H, Me-C=), 3.75 (d, *J =* 11.2 Hz, 3H, MeO). ^13^C-NMR (150.9 MHz) δ = 13.9, 19.1 (*J =* 6.6 Hz), 20.6, 22.5, 25.4, 30.0, 30.4 (*J =* 8.2 Hz), 31.4, 32.9, 53.1 (*J =* 6.7 Hz), 63.3, 66.4 (*J =* 10.3 Hz), 92.7, 98.5 (*J =* 190.4 Hz), 104.2 (*J =* 15.3 Hz), 207.4 (*J =* 5.1 Hz). ^3^^1^P-NMR (242.9 MHz): δ 22.2. Anal. Calcd for C_18_H_33_O_5_P (360.43): C 59.98, H 9.23. Found: C 60.05, H 9.29.

### 3.5. General Procedure for Synthesis of the 2-(2-Diphenylphosphinoyl-2,3-dienyloxy)-tetrahydro-2H-pyrans **9**

To a solution of the (tetrahydro-2H-pyran-2-yloxy)-alkynols **5** (20.0 mmol) and triethylamine (2.2 g, 22.0 mmol) [0.037 g/mL] in dry diethyl ether (60 mL) at −70 °C a solution of freshly distilled diphenylchlorophosphine (4.4 g, 20.0 mmol) [0.22 g/mL] in the same solvent (20 mL) was added dropwise with stirring. The reaction mixture was stirred for an hour at the same temperature and for 8 h at room temperature and then washed with water, 2 N HCl, extracted with diethyl ether, and the extract was washed with saturated NaCl, and dried over anhydrous sodium sulfate. The solvent was removed using a rotatory evaporator and the residue was purified by column chromatography on a silica gel (Kieselgel Merck 60 F_254_) with ethyl acetate-hexane (10:1) to give the pure products **9** as oils, which had the following properties:

*2-(2-Diphenylphosphinoyl-4-methyl-hexa-2,3-dienyloxy)-tetrahydro-2H-pyran* (**9a**). Yellow oil, yield: 86%. R_f_ 0.58; IR (neat, cm^−1^): 1119 (C-O-C), 1157 (P=O), 1437, 1483 (Ph), 1949 (C=C=C). ^1^H-NMR (600.1 MHz): δ 0.75 (t, *J =* 7.4 Hz, 3H, Me-CH_2_), 1.27–1.82, 3.71–3.77, 4.59–4.62 (overlapping multiplets, 9H, OTHP), 1.52 (d, *J =* 6.2 Hz, 3H, Me-C=), 2.05 (m, 2H, Me-CH_2_), 4.26–4.53 (m, 2H, CH_2_O), 7.41–7.78 (m, 10H, 2Ph). ^13^C-NMR (150.9 MHz) δ = 11.8, 17.6 (*J =* 5.6 Hz), 18.9, 25.4, 26.3, 30.1, 61.6, 64.2 (*J =* 9.5 Hz), 95.9 (*J =* 104.4 Hz), 97.6, 104.6 (*J =* 13.9 Hz), 131.7–133.8 (2Ph), 208.2 (*J =* 6.5 Hz). ^31^P-NMR (242.9 MHz): δ 29.5. Anal. Calcd for C_24_H_29_O_3_P (396.46): C 72.71, H 7.37. Found: C 72.63, H 7.42.

*2-(2-Diphenylphosphinoyl-4-methyl-octa-2,3-dienyloxy)-tetrahydro-2H-pyran* (**9b**). Yellow oil, yield: 84%. R_f_ 0.57; IR (neat, cm^−1^): 1120 (C-O-C), 1155 (P=O), 1438, 1482 (Ph), 1954 (C=C=C). ^1^H-NMR (600.1 MHz): δ 0.81 (t, *J =* 7.3 Hz, 3H, Me-CH_2_), 1.07–1.18, 3.41–3.45 (mm, 6H, (CH_2_)_3_-Me), 1.34–1.74, 3.71–3.77, 4.58–4.61 (overlapping multiplets, 9H, OTHP), 1.51 (d, *J =* 6.6 Hz, 3H, Me-C=), 4.25–4.52 (m, 2H, CH_2_O), 7.30–7.82 (m, 10H, 2Ph). ^13^C-NMR (150.9 MHz) δ = 13.9, 17.7 (*J =* 5.6 Hz), 18.9, 22.2, 25.4, 30.1, 29.2, 32.8, 61.7, 64.3 (*J =* 9.6 Hz), 95.2 (*J =* 104.5 Hz), 97.8, 103.0 (*J =* 13.3 Hz), 131.5–133.4 (2Ph), 208.5 (*J =* 6.4 Hz). ^31^P-NMR (242.9 MHz): δ 29.8. Anal. Calcd for C_26_H_33_O_3_P (424.51): C 73.56, H 7.84. Found: C 73.64, H 7.91. 

*2-(3-Cyclohexylidene-2-diphenylphosphinoyl-allyloxy)-tetrahydro-2H-pyran* (**9c**). Yellow oil, yield: 81%. R_f_ 0.56; IR (neat, cm^−1^): 1123 (C-O-C), 1169 (P=O), 1436, 1490 (Ph), 1954 (C=C=C). ^1^H-NMR (600.1 MHz): δ 0.97–1.06, 1.86–2.02, 3.40–3.44 (overlapping multiplets, 10H, (CH_2_)_5_), 1.27–1.57, 3.72–3.77, 4.58–4.60 (overlapping multiplets, 9H, OTHP), 4.29–4.51 (m, 2H, CH_2_O), 7.26–7.78 (m, 10H, 2Ph). ^13^C-NMR (150.9 MHz) δ = 18.9, 21.1, 25.4, 26.3 (*J =* 3.8 Hz), 29.9 (*J =* 5.2 Hz), 30.1, 61.8, 64.1 (*J =* 9.6 Hz), 94.0 (*J =* 105.2 Hz), 97.5, 104.9 (*J =* 13.4 Hz), 128.1–133.0 (2Ph), 205.4 (*J =* 6.8 Hz). ^31^P-NMR (242.9 MHz): δ 31.1. Anal. Calcd for C_26_H_31_O_3_P (422.50: C 73.91, H 7.40. Found: C 73.83, H 7.31.

*2-(2-Diphenylphosphinoyl-1,4-dimethyl-hexa-2,3-dienyloxy)-tetrahydro-2H-pyran* (**9d**). Orange oil, yield: 83%. R_f_ 0.46; IR (neat, cm^−1^): 1119 (C-O-C), 1158 (P=O), 1440, 1489 (Ph), 1950 (C=C=C). ^1^H-NMR (600.1 MHz): δ 0.84 (t, *J =* 7.3 Hz, 3H, Me-CH_2_), 1.30–1.71, 3.61–3.65, 4.56–4.59 (overlapping multiplets, 9H, OTHP), 1.43 (dd, *J =* 6.3 Hz, *J =* 9.8 Hz, 3H, Me-CHO), 1.53 (d, *J =* 6.4 Hz, 3H, Me-C=), 2.02 (m, 2H, Me-CH_2_), 4.61–4.67 (m, 1H, CHO), 7.29–7.82 (m, 10H, 2Ph). ^13^C-NMR (150.9 MHz) δ = 12.7, 18.6 (*J =* 5.5 Hz), 19.5, 22.5 (*J =* 7.7 Hz), 22.6, 27.5 (*J =* 5.4 Hz), 30.6, 62.4, 64.9 (*J =* 9.4 Hz), 97.6 (*J =* 104.1 Hz), 96.7, 104.7 (*J =* 13.7 Hz), 129.2–134.5 (2Ph), 204.7 (*J =* 6.6 Hz). ^31^P-NMR (242.9 MHz): δ 30.4. Anal. Calcd for C_25_H_31_O_3_P (410.49): C 73.15, H 7.61. Found: C 73.08, H 7.69.

*2-(2-Diphenylphosphinoyl-1,4-dimethyl-octa-2,3-dienyloxy)-tetrahydro-2H-pyran* (**9e**). Orange oil, yield: 82%. R_f_ 0.45; IR (neat, cm^−1^): 1123 (C-O-C), 1165 (P=O), 1437, 1492 (Ph), 1954 (C=C=C). ^1^H-NMR (600.1 MHz): δ 0.81 (t, *J =* 7.5 Hz, 3H, Me-CH_2_), 1.10–1.21, 3.50–3.55 (mm, 6H, (CH_2_)_3_-Me), 1.37–1.71, 3.62–3.67, 4.57–4.63 (overlapping multiplets, 9H, OTHP), 1.42 (dd, *J =* 6.4 Hz, *J =* 9.7 Hz, 3H, Me-CHO), 1.55 (d, *J =* 6.3 Hz, 3H, Me-C=), 4.52-4.57 (m, 1H, CHO), 7.28-7.84 (m, 10H, 2Ph). ^13^C-NMR (150.9 MHz) δ = 13.8, 18.4 (*J =* 5.6 Hz), 19.6, 21.3, 22.2 (*J =* 7.5 Hz), 25.5, 30.5, 29.5, 32.9, 62.7, 65.2 (*J =* 9.7 Hz), 97.4, 97.9 (*J =* 105.0 Hz), 104.7 (*J =* 13.7 Hz), 129.7–134.6 (2Ph), 207.7 (*J =* 6.6 Hz). ^31^P-NMR (242.9 MHz): δ 29.7. Anal. Calcd for C_27_H_35_O_3_P (438.54): C 73.95, H 8.04. Found: C 74.03, H 7.99.

*2-(3-Cyclohexylidene-2-diphenylphosphinoyl-1-methyl-allyloxy)-tetrahydro-2H-pyran* (**9f**). Yellow oil, yield: 80%. R_f_ 0.45; IR (neat, cm^−1^): 1118 (C-O-C), 1160 (P=O), 1439, 1488 (Ph), 1949 (C=C=C). ^1^H-NMR (600.1 MHz): δ 1.03–1.11, 1.91–1.97, 3.33–3.45 (overlapping multiplets, 10H, (CH_2_)_5_), 1.31–1.62, 3.68–3.79, 4.56–4.70 (overlapping multiplets, 9H, OTHP), 1.44 (d, *J =* 6.5 Hz, 3H, Me-CHO), 4.51–4.57 (m, 1H, CHO), 7.31–7.87 (m, 10H, 2Ph). ^13^C-NMR (150.9 MHz) δ = 20.0, 20.7, 21.7 (*J =* 7.4 Hz), 26.1, 26.7 (*J =* 3.6 Hz), 30.2, 30.4 (*J =* 5.3 Hz), 62.8, 67.8 (*J =* 9.6 Hz), 97.7, 99.8 (*J =* 105.0 Hz), 106.3 (*J =* 13.8 Hz), 127.7–134.2 (2Ph), 203.6 (*J =* 7.2 Hz). ^31^P-NMR (242.9 MHz): δ 31.2. Anal. Calcd for C_27_H_33_O_3_P (436.52): C 74.29, H 7.62. Found: C 74.33, H 7.69.

*2-(2-Diphenylphosphinoyl-1,1,4-trimethyl-hexa-2,3-dienyloxy)-tetrahydro-2H-pyran* (**9g**). Dark orange oil, yield: 80%. R_f_ 0.44; IR (neat, cm^−1^): 1119 (C-O-C), 1154 (P=O), 1436, 1487 (Ph), 1956 (C=C=C). ^1^H-NMR (600.1 MHz): δ 1.03 (t, *J =* 7.5 Hz, 3H, Me-CH_2_), 1.38–1.69, 3.53–3.73, 4.61–4.77 (overlapping multiplets, 9H, OTHP), 1.47 (d, *J =* 10.6 Hz, 6H, Me_2_CO), 1.53 (d, *J =* 6.5 Hz, 3H, Me-C=), 2.02 (m, 2H, Me-CH_2_), 7.41–7.85 (m, 10H, 2Ph). ^13^C-NMR (150.9 MHz) δ = 12.1, 18.5 (*J =* 5.7 Hz), 19.4, 26.2, 28.4 (*J =* 5.5 Hz), 31.1, 31.2 (*J =* 8.0 Hz), 63.0, 68.4 (*J =* 9.7 Hz), 96.9, 97.8 (*J =* 104.7 Hz), 105.1 (*J =* 13.4 Hz), 127.4–133.9 (2Ph), 204.5 (*J =* 7.0 Hz). ^31^P-NMR (242.9 MHz): δ 31.7. Anal. Calcd for C_26_H_33_O_3_P (424.51): C 73.56, H 7.84. Found: C 73.63, H 7.92.

*2-(2-Diphenylphosphinoyl-1,1,4-trimethyl-octa-2,3-dienyloxy)-tetrahydro-2H-pyran* (**9h**). Yellow oil, yield: 78%. R_f_ 0.45; IR (neat, cm^−1^): 1119 (C-O-C), 1162 (P=O), 1440, 1486 (Ph), 1953 (C=C=C). ^1^H-NMR (600.1 MHz): δ 1.06 (t, *J =* 7.6 Hz, 3H, Me-CH_2_), 1.09–1.22, 3.43–3.46 (mm, 6H, (CH_2_)_3_-Me), 1.29–1.64, 3.57–3.74, 4.59–4.74 (overlapping multiplets, 9H, OTHP), 1.50 (d, *J =* 10.5 Hz, 3H, Me_2_CO), 1.55 (d, *J =* 6.6 Hz, 3H, Me-C=), 7.37–7.84 (m, 10H, 2Ph). ^13^C-NMR (150.9 MHz) δ = 13.8, 18.1 (*J =* 5.7 Hz), 18.7, 21.7, 25.8, 30.0, 30.4, 30.7 (*J =* 8.2 Hz), 33.1, 62.0, 67.9 (*J =* 9.5 Hz), 97.3, 98.4 (*J =* 105.3 Hz), 104.8 (*J =* 13.5 Hz), 128.0–134.4 (2Ph), 205.4 (*J =* 7.2 Hz). ^31^P-NMR (242.9 MHz): δ 30.6. Anal. Calcd for C_28_H_37_O_3_P (452.57): C 74.31, H 8.24. Found: C 74.24, H 8.17.

### 3.6. General Procedure for Synthesis of the 1-Hydroxyalkyl-1,2-dienephosphonates **10**, the 3-Diphenylphosphinoyl-2,3-dien-1-ols **11a–c** and the 3-Diphenylphosphinoyl-3,4-dien-2-ols **11d–h**

A solution of the dimethyl 1-(tetrahydro-2*H*-pyran-2-yloxy)-1,2-dienephosphonates **7** or the 2-(2-diphenylphosphinoyl-2,3-dienyloxy)-tetrahydro-2*H*-pyrans **9** (5.0 mmol) and PPTS (1.13 g, 0.5 mmol) [0.113 g/mL] in ethanol (10 mL) was stirred at room temperature for 6 h. The mixture was then washed with water, extracted with methylene chloride and dried over anhydrous sodium sulfate. After evaporation of the solvent, the residue was chromatographied on a column (silica gel, Kieselgel Merck 60 F_254_) with a mixture of ethyl acetate and hexane (10:1) as a eluent to give the pure products **10** or **11** as oils, which had the following properties:

*Dimethyl 1-hydroxymethyl-3-methylpenta-1,2-dienephosphonate* (**10a**). Pale yellow oil, yield: 80%. R_f_ 0.45; IR (neat, cm^−1^): 1248 (P=O), 1956 (C=C=C), 3404 (OH). ^1^H-NMR (250.1 MHz): δ 1.06 (t, *J =* 7.4 Hz, 3H, Me-CH_2_), 1.80 (d, *J =* 6.7 Hz, 3H, Me-C=), 2.04–2.12 (m, 2H, Me-CH_2_), 2.64 (s, 1H, OH), 3.75 (d, *J =* 11.8 Hz, 3H, MeO), 4.30–4.36 (m, 2H, CH_2_O). ^13^C-NMR (62.9 MHz) δ = 12.0 (*J =* 7.7 Hz), 18.1 (*J =* 6.5 Hz), 26.5 (*J =* 9.3 Hz), 52.8 (*J =* 6.3 Hz), 64.9 (*J =* 10.1 Hz), 90.8 (*J =* 191.3 Hz), 104.7 (*J =* 15.7 Hz), 208.7 (*J =* 5.6 Hz). ^31^P-NMR (101.2 MHz): δ 21.6. Anal. Calcd for C_9_H_17_O_4_P (220.20): C 49.09, H 7.78. Found: C 49.17, H 7.71.

*Dimethyl 1-hydroxymethyl-3-methylhepta-1,2-dienephosphonate* (**10b**). Pale yellow oil, yield: 78%. R_f_ 0.43; IR (neat, cm^−1^): 1249 (P=O), 1958 (C=C=C), 3401 (OH). ^1^H-NMR (600.1 MHz): δ 0.99 (t, *J =* 7.3 Hz, 3H, Me-CH_2_), 1.32–1.46, 1.51–1.63, 2.03–2.09 (overlapping multiplets, 10H, Me-(CH_2_)_3_), 1.79 (d, *J =* 6.5 Hz, 3H, Me-C=), 2.64 (s, 1H, OH), 3.76 (d, *J =* 11.2 Hz, 3H, MeO), 4.33–4.38 (m, 2H, CH_2_O). ^13^C-NMR (150.9 MHz) δ = 13.9, 18.1 (*J =* 6.6 Hz), 22.2, 30.3, 32.9, 52.8 (*J =* 6.2 Hz), 64.9 (*J =* 10.1 Hz), 90.4 (*J =* 191.5 Hz), 103.7 (*J =* 15.6 Hz), 208.8 (*J =* 5.5 Hz). ^31^P-NMR (242.9 MHz): δ 21.0. Anal. Calcd for C_11_H_21_O_4_P (248.26): C 53.22, H 8.53. Found: C 53.30, H 8.62.

*Dimethyl 2-cyclohexylidene-1-hydroxymethyl-ethenephosphonate* (**10c**). Colourless oil, yield: 77%. R_f_ 0.44; IR (neat, cm^−1^): 1259 (P=O), 1952 (C=C=C), 3412 (OH). ^1^H-NMR (250.1 MHz): δ 1.22–1.37, 1.80–1.96, 3.49–3.57 (overlapping multiplets, 10H, (CH_2_)_5_), 2.67 (s, 1H, OH), 3.75 (d, *J =* 11.3 Hz, 3H, MeO), 4.23–4.29 (m, 2H, CH_2_O). ^13^C-NMR (62.9 MHz) δ = 25.6, 27.1, 30.4 (*J =* 5.8 Hz), 52.8 (*J =* 6.0 Hz), 64.7 (*J =* 10.7 Hz), 88.8 (*J =* 190.3 Hz), 105.1 (*J =* 15.3 Hz), 205.6 (*J =* 5.4 Hz). ^31^P-NMR (101.2 MHz): δ 20.8. Anal. Calcd for C_11_H_19_O_4_P (246.24): C 53.65, H 7.78. Found: C 53.72, H 7.73.

*Dimethyl 1-(1-hydroxyethyl)-3-methylpenta-1,2-dienephosphonate* (**10d**). Yellow oil, yield: 80%. R_f_ 0.58; IR (neat, cm^−1^): 1254 (P=O), 1956 (C=C=C), 3372 (OH). ^1^H-NMR (600.1 MHz): δ 0.98 (t, *J =* 7.5 Hz, 3H, Me-CH_2_), 1.42 (dd, *J =* 6.1 Hz, *J =* 10.2 Hz, 3H, Me-CHO), 1.78 (d, *J =* 6.6 Hz, 3H, Me-C=), 2.02–2.10 (m, 2H, Me-CH_2_), 2.70 (s, 1H, OH), 3.78 (d, *J =* 11.6 Hz, 3H, MeO), 4.67–4.72 (m, 1H, Me-CHO). ^13^C-NMR (150.9 MHz) δ = 12.2 (*J =* 7.6 Hz), 18.4 (*J =* 6.4 Hz), 23.2 (*J =* 7.5 Hz), 27.4 (*J =* 9.2 Hz), 52.6 (*J =* 6.2 Hz), 66.9 (*J =* 10.3 Hz), 96.3 (*J =* 192.3 Hz), 104.4 (*J =* 15.9 Hz), 208.9 (*J =* 5.4 Hz). ^31^P-NMR (242.9 MHz): δ 21.1. Anal. Calcd for C_10_H_19_O_4_P (234.23): C 51.28, H 8.18. Found: C 51.21, H 8.13.

*Dimethyl 1-(1-hydroxyethyl)-3-methylhepta-1,2-dienephosphonate* (**10e**). Yellow oil, yield: 79%. R_f_ 0.57; IR (neat, cm^−1^): 1248 (P=O), 1958 (C=C=C), 3437 (OH). ^1^H-NMR (600.1 MHz): δ 1.09 (t, *J =* 7.4 Hz, 3H, Me-CH_2_), 1.39–1.44, 1.50–1.55, 2.11–2.15 (overlapping multiplets, 10H, Me-(CH_2_)_3_), 1.40 (dd, *J =* 6.3 Hz, *J =* 10.3 Hz, 3H, Me-CHO), 1.77 (d, *J =* 6.9 Hz, 3H, Me-C=), 2.68 (s, 1H, OH), 3.77 (d, *J =* 11.5 Hz, 3H, MeO), 4.50–4.55 (m, 1H, Me-CHO). ^13^C-NMR (150.9 MHz) δ = 13.7, 18.7 (*J =* 6.4 Hz), 23.0, 23.5 (*J =* 7.5 Hz), 30.0, 33.0, 52.3 (*J =* 6.2 Hz), 68.7 (*J =* 10.0 Hz), 91.5 (*J =* 191.5 Hz), 103.2 (*J =* 16.1 Hz), 208.7 (*J =* 5.4 Hz). ^31^P-NMR (242.9 MHz): δ 21.2. Anal. Calcd for C_12_H_23_O_4_P (262.28): C 54.95, H 8.84. Found: C 55.02, H 8.78.

*Dimethyl 1-cyclohexylidenemethylene-2-hydroxypropanephosphonate* (**10f**). Orange oil, yield: 81%. R_f _0.59; IR (neat, cm^−1^): 1253 (P=O), 1951 (C=C=C), 3422 (OH). ^1^H-NMR (600.1 MHz): δ 1.33–1.48, 1.87–2.00, 3.12–3.20 (overlapping multiplets, 10H, (CH_2_)_5_), 1.38 (dd, *J =* 6.4 Hz, *J =* 9.7 Hz, 3H, Me-CHO), 2.84 (s, 1H, OH), 3.78 (d, *J =* 11.6 Hz, 3H, MeO), 4.64–4.69 (m, 1H, Me-CHO). ^13^C-NMR (150.9 MHz) δ = 23.3 (*J =* 7.3 Hz), 25.7, 27.0, 30.3 (*J =* 6.2 Hz), 53.1 (*J =* 6.1 Hz), 65.9 (*J =* 10.0 Hz), 94.7 (*J =* 186.1 Hz), 106.8 (*J =* 15.5 Hz), 202.3 (*J =* 5.1 Hz). ^31^P-NMR (242.9 MHz): δ 20.9. Anal. Calcd for C_12_H_21_O_4_P (260.27): C 55.38, H 8.31. Found: C 55.45, H 8.26.

*Dimethyl 1-(1-hydroxy-1-methylethyl)-3-methylpenta-1,2-dienephosphonate* (**10g**). Yellow oil, yield: 79%. R_f_ 0.60; IR (neat, cm^−1^): 1250 (P=O), 1953 (C=C=C), 3398 (OH). ^1^H-NMR (600.1 MHz): δ 1.11 (t, *J =* 7.6 Hz, 3H, Me-CH_2_), 1.54 (d, *J =* 10.7 Hz, 3H, Me_2_CO), 1.75 (d, *J =* 6.7 Hz, 3H, Me-C=), 2.04–2.13 (m, 2H, Me-CH_2_), 2.93 (s, 1H, OH), 3.79 (d, *J =* 11.5 Hz, 3H, MeO). ^13^C-NMR (150.9 MHz) δ = 12.3, 18.2 (*J =* 6.5 Hz), 27.4 (*J =* 9.2 Hz), 31.0 (*J =* 8.2 Hz), 53.0 (*J =* 6.6 Hz), 68.2 (*J =* 10.2 Hz), 99.5 (*J =* 190.2 Hz), 104.3 (*J =* 15.4 Hz), 207.4 (*J =* 5.2 Hz). ^3^^1^P-NMR (242.9 MHz): δ 22.4. Anal. Calcd for C_11_H_21_O_4_P (248.26): C 53.22, H 8.53. Found: C 53.15, H 8.44.

*Dimethyl 1-(1-hydroxy-1-methylethyl)-3-methylhepta-1,2-dienephosphonate* (**10h**). Orange oil, yield: 78%. R_f_ 0.57; IR (neat, cm^−1^): 1255 (P=O), 1954 (C=C=C), 3416 (OH). ^1^H-NMR (600.1 MHz): δ 0.92 (t, *J =* 7.3 Hz, 3H, Me-CH_2_), 1.28–1.40, 1.53–1.66, 2.05–2.13 (overlapping multiplets, 10H, Me-(CH_2_)_3_), 1.55 (d, *J =* 10.8 Hz, 3H, Me_2_CO), 1.75 (d, *J =* 6.7 Hz, 3H, Me-C=), 2.95 (s, 1H, OH), 3.75 (d, *J =* 11.4 Hz, 3H, MeO). ^13^C-NMR (150.9 MHz) δ = 14.0, 19.0 (*J =* 6.7 Hz), 22.7, 29.8, 31.3 (*J =* 8.2 Hz), 33.1, 53.0 (*J =* 6.7 Hz), 66.6 (*J =* 10.3 Hz), 99.7 (*J =* 182.2 Hz), 104.1 (*J =* 15.7 Hz), 207.3 (*J =* 5.1 Hz). ^3^^1^P-NMR (242.9 MHz): δ 22.8. Anal. Calcd for C_13_H_25_O_4_P (276.31): C 56.51, H 9.12. Found: C 56.59, H 9.06.

*2-Diphenylphosphinoyl-4-methylhexa-2,3-dien-1-ol* (**11a**). Colourless oil, yield: 86%. R_f_ 0.42; IR (neat, cm^−1^): 1175 (P=O), 1440, 1489 (Ph), 1955 (C=C=C), 3378 (OH). ^1^H-NMR (600.1 MHz): δ 0.72 (t, *J =* 7.4 Hz, 3H, Me-CH_2_), 1.54 (d, *J =* 6.0 Hz, 3H, Me-C=), 1.66–1.88 (m, 2H, Me-CH_2_), 2.66 (s, 1H, OH), 4.41–4.47 (m, 2H, CH_2_O), 7.28–7.82 (m, 10H, 2Ph). ^13^C-NMR (150.9 MHz) δ = 11.7, 17.6 (*J =* 5.6 Hz), 26.4, 64.2 (*J =* 7.5 Hz), 97.5 (*J =* 103.8 Hz), 105.3 (*J =* 13.6 Hz), 128.2–132.5 (2Ph), 206.3 (*J =* 7.2 Hz). ^31^P-NMR (242.9 MHz): δ 33.5. Anal. Calcd for C_19_H_21_O_2_P (312.34): C 73.06, H 6.78. Found: C 73.14, H 6.71.

*2-Diphenylphosphinoyl-4-methylocta-2,3-dien-1-ol* (**11b**). Yellow oil, yield: 83%. R_f_ 0.41; IR (neat, cm^−1^): 1177 (P=O), 1436, 1492 (Ph), 1950 (C=C=C), 3374 (OH). ^1^H-NMR (600.1 MHz): δ 0.81 (t, *J =* 7.2 Hz, 3H, Me-CH_2_), 1.04–1.17, 1.34–1.50, 1.67–1.84 (overlapping multiplets, 10H, Me-(CH_2_)_3_), 1.53 (d, *J =* 6.2 Hz, 3H, Me-C=), 2.65 (s, 1H, OH), 4.39–4.46 (m, 2H, CH_2_O), 7.30–7.80 (m, 10H, 2Ph). ^13^C-NMR (150.9 MHz) δ = 13.8, 17.6 (*J =* 5.4 Hz), 18.8, 29.2, 32.9, 64.3 (*J =* 7.6 Hz), 96.7 (*J =* 103.9 Hz), 103.6 (*J =* 13.5 Hz), 128.7–132.5 (2Ph), 206.5 (*J =* 7.2 Hz). ^31^P-NMR (242.9 MHz): δ 32.9. Anal. Calcd for C_21_H_25_O_2_P (340.40): C 74.10, H 7.40. Found: C 74.17, H 7.32.

*3-Cyclohexylidene-2-diphenylphosphinoylprop-2-en-1-ol* (**11c**). Pale yellow oil, yield: 81%. R_f_ 0.41; IR (neat, cm^−1^): 1170 (P=O), 1439, 1488 (Ph), 1947 (C=C=C), 3387 (OH). ^1^H-NMR (250.1 MHz): δ 0.97–1.04, 1.89–2.04, 3.38–3.54 (overlapping multiplets, 10H, (CH_2_)_5_), 2.64 (s, 1H, OH), 4.38–4.43 (m, 2H, CH_2_O), 7.28–7.79 (m, 10H, 2Ph). ^13^C-NMR (62.9 MHz) δ = 25.4, 26.4, 30.0, 62.1 (*J =* 7.6 Hz), 95.6 (*J =* 104.1 Hz), 105.4 (*J =* 13.3 Hz), 128.8–132.5 (2Ph), 203.6 (*J =* 7.3 Hz). ^31^P-NMR (101.2 MHz): δ 33.3. Anal. Calcd for C_21_H_23_O_2_P (338.38): C 74.54, H 6.85. Found: C 74.62, H 6.79.

*3-Diphenylphosphinoyl-5-methtlhepta-3,4-dien-2-ol* (**11d**). Light orange oil, yield: 87%. R_f_ 0.59; IR (neat, cm^−1^): 1174 (P=O), 1441, 1490 (Ph), 1951 (C=C=C), 3369 (OH). ^1^H-NMR (600.1 MHz): δ 0.86 (t, *J =* 7.4 Hz, 3H, Me-CH_2_), 1.35 (dd, *J =* 6.2 Hz, *J =* 9.4 Hz, 3H, Me-CHO), 1.58 (d, *J =* 6.3 Hz, 3H, Me-C=), 1.78–1.90 (m, 2H, Me-CH_2_), 2.70 (s, 1H, OH), 4.59–4.63 (m, 1H, Me-CHO), 7.35–7.90 (m, 10H, 2Ph). ^13^C-NMR (150.9 MHz) δ = 12.4, 18.5 (*J =* 5.4 Hz), 22.4 (*J =* 7.6 Hz), 26.7, 64.2 (*J =* 7.4 Hz), 96.5 (*J =* 104.2 Hz), 105.1 (*J =* 13.4 Hz), 129.1–132.4 (2Ph), 204.1 (*J =* 7.1 Hz). ^31^P-NMR (242.9 MHz): δ 34.2. Anal. Calcd for C_20_H_23_O_2_P (326.37): C 73.60, H 7.10. Found: C 73.67, H 7.05.

*3-Diphenylphosphinoyl-5-methylnona-3,4-dien-2-ol* (**11e**). Yellow oil, yield: 85%. R_f_ 0.61; IR (neat, cm^−1^): 1168 (P=O), 1438, 1487 (Ph), 1952 (C=C=C), 3379 (OH). ^1^H-NMR (600.1 MHz): δ 0.92 (t, *J =* 7.3 Hz, 3H, Me-CH_2_), 1.11–1.23, 1.29–1.47, 1.69–1.96 (overlapping multiplets, 10H, Me-(CH_2_)_3_), 1.37 (dd, *J =* 6.3 Hz, *J =* 9.6 Hz, 3H, Me-CHO), 1.56 (d, *J =* 6.4 Hz, 3H, Me-C=), 2.72 (s, 1H, OH), 4.61–4.67 (m, 1H, Me-CHO), 7.39–7.89 (m, 10H, 2Ph). ^13^C-NMR (150.9 MHz) δ = 13.7, 18.3 (*J =* 5.5 Hz), 18.9, 22.3 (*J =* 7.7 Hz), 29.5, 33.2, 65.4 (*J =* 7.6 Hz), 100.7 (*J =* 103.8 Hz), 104.8 (*J =* 13.5 Hz), 128.4–132.5 (2Ph), 205.3 (*J =* 7.3 Hz). ^31^P-NMR (242.9 MHz): δ 34.5. Anal. Calcd for C_22_H_27_O_2_P (354.42): C 74.55, H 7.68. Found: C 74.61, H 7.60.

*4-Cyclohexylidene-3-diphenylphosphinoylbut-3-en-2-ol* (**11f**). Yellow oil, yield: 88%. R_f_ 0.58; IR (neat, cm^−1^): 1168 (P=O), 1436, 1493 (Ph), 1948 (C=C=C), 3395 (OH). ^1^H-NMR (600.1 MHz): δ 0.99–1.07, 1.84–2.01, 3.37–3.57 (overlapping multiplets, 10H, (CH_2_)_5_), 1.34 (dd, *J =* 6.2 Hz, *J =* 9.4 Hz, 3H, Me-CHO), 2.73 (s, 1H, OH), 4.64–4.69 (m, 1H, Me-CHO), 7.32–7.84 (m, 10H, 2Ph). ^13^C-NMR (150.9 MHz) δ = 22.2 (*J =* 7.5 Hz), 25.5, 26.5, 30.0, 66.2 (*J =* 7.3 Hz), 100.2 (*J =* 105.0 Hz), 106.6 (*J =* 13.6 Hz), 128.1–132.5 (2Ph), 202.6 (*J =* 7.4 Hz). ^31^P-NMR (242.9 MHz): δ 33.9. Anal. Calcd for C_22_H_25_O_2_P (352.41): C 74.98, H 7.15. Found: C 75.05, H 7.09.

*3-Diphenylphosphinoyl-2,5-dimethylhepta-3,4-dien-2-ol* (**11g**). Orange oil, yield: 84%. R_f_ 0.60; IR (neat, cm^−1^): 1171 (P=O), 1437, 1488 (Ph), 1954 (C=C=C), 3373 (OH). ^1^H-NMR (600.1 MHz): δ 1.09 (t, *J =* 7.3 Hz, 3H, Me-CH_2_), 1.49 (d, *J =* 10.1 Hz, 3H, Me_2_CO), 1.53 (d, *J =* 6.4 Hz, 3H, Me-C=), 1.81–1.86 (m, 2H, Me-CH_2_), 2.74 (s, 1H, OH), 7.28–7.88 (m, 10H, 2Ph). ^13^C-NMR (150.9 MHz) δ = 12.3, 18.4 (*J =* 5.6 Hz), 27.2, 31.4 (*J =* 8.1 Hz), 67.0 (*J =* 7.4 Hz), 98.3 (*J =* 104.8 Hz), 105.3 (*J =* 13.5 Hz), 128.3–132.4 (2Ph), 204.7 (*J =* 7.2 Hz). ^3^^1^P-NMR (242.9 MHz): δ 33.8. Anal. Calcd for C_21_H_25_O_2_P (340.40): C 74.10, H 7.40. Found: C 74.01, H 7.45.

*3-Diphenylphosphinoyl-2,5-dimethylnona-3,4-dien-2-ol* (**11h**). Dark orange oil, yield: 83%. R_f_ 0.56; IR (neat, cm^−1^): 1165 (P=O), 1439, 1486 (Ph), 1955 (C=C=C), 3394 (OH). ^1^H-NMR (600.1 MHz): δ 1.07 (t, *J =* 7.3 Hz, 3H, Me-CH_2_), 1.12–1.25, 1.32–1.45, 1.73–1.89 (overlapping multiplets, 10H, Me-(CH_2_)_3_), 1.50 (d, *J =* 10.0 Hz, 3H, Me_2_CO), 1.55 (d, *J =* 6.3 Hz, 3H, Me-C=), 2.73 (s, 1H, OH), 7.29–7.90 (m, 10H, 2Ph). ^13^C-NMR (150.9 MHz) δ = 13.9, 18.2 (*J =* 5.7 Hz), 19.0, 30.1, 31.6 (*J =* 8.2 Hz), 33.2, 68.1 (*J =* 7.5 Hz), 98.7 (*J =* 105.0 Hz), 105.0 (*J =* 13.4 Hz), 128.4–132.6 (2Ph), 205.1 (*J =* 7.3 Hz). ^31^P-NMR (242.9 MHz): δ 34.1. Anal. Calcd for C_23_H_29_O_2_P (368.45): C 74.98, H 7.93. Found: C 74.92, H 8.01.

## 4. Conclusions

In conclusion, a convenient and efficient method for regioselective synthesis of a new family of 1,1-bifunctionalized allenes has been explored. Phosphorylated α-hydroxyallenes prepared were derived from [2,3]-sigmatropic rearrangement of the intermediate propargyl phosphites or phosphinites formed in the reaction of protected alkynols with dimethylchloro phosphite or chlorodiphenyl phosphine in the presence of a base. Further investigations on this potentially important synthetic methodology are currently in progress. At the same time, the synthetic application of the prepared phosphorylated α-hydroxyallenes with protected or unprotected hydroxy group for synthesis of different heterocyclic compounds is now under investigation in our laboratory as a part of our general synthetic strategy for investigation of the scope and limitations of the electrophilic cyclization and cycloisomerization reactions of bifunctionalized allenes. Results of these investigations will be reported in due course.
